# Phlebotomine (Diptera: Psychodidae) species and their blood meal sources in a new leishmaniasis focus in Los Montes de María, Bolívar, in northern Colombia

**DOI:** 10.7705/biomedica.6876

**Published:** 2024-05-30

**Authors:** Yeisson Cera-Vallejo, Marlon Mauricio Ardila, Leidi Herrera, Lina Martínez, Alveiro Pérez-Doria

**Affiliations:** 1 Programa de Licenciatura en Biología y Química, Facultad de Educación, Universidad del Atlántico, Puerto Colombia, Colombia Universidad del Atlántico Facultad de Educación Universidad del Atlántico Puerto Colombia Colombia; 2 Departamento de Patología y Medicina Preventiva, Facultad de Ciencias Veterinarias, Universidad de Concepción, Chillán, Chile Universidad de Concepción Facultad de Ciencias Veterinarias Universidad de Concepción Chillán Chile; 3 Grupo Interdisciplinario en Ciencias Marinas y Ambientales (GICMARA), Facultad de Ciencias Básicas, Universidad del Atlántico, Puerto Colombia, Colombia Universidad del Atlántico Facultad de Ciencias Básicas Universidad del Atlántico Puerto Colombia Colombia; 4 Instituto de Zoología y Ecología Tropical, Facultad de Ciencias, Universidad Central de Venezuela, Caracas, Venezuela Universidad Central de Venezuela Instituto de Zoología y Ecología Tropical Facultad de Ciencias Universidad Central de Venezuela Caracas Venezuela; 5 Instituto de Investigaciones en Ciencias de la Salud, Universidad Nacional de Asunción, San Lorenzo, Paraguay Universidad Nacional de Asunción Instituto de Investigaciones en Ciencias de la Salud Universidad Nacional de Asunción San Lorenzo Paraguay; 6 Grupo de Investigaciones Biomédicas, Facultad de Educación y Ciencias, Universidad de Sucre, Sincelejo, Colombia Universidad de Sucre Grupo de Investigaciones Biomédicas Facultad de Educación y Ciencias Universidad de Sucre Sincelejo Colombia; 7 Facultad de Medicina Veterinaria y Zootecnia, Departamento de Ciencias Pecuarias, Programa de Doctorado en Microbiología y Salud Tropical, Universidad de Córdoba, Montería, Colombia Universidad de Córdoba Facultad de Medicina Veterinaria y Zootecnia Departamento de Ciencias Pecuarias Universidad de Córdoba Montería Colombia; 8 División de Investigación, Innovación y Desarrollo, Pyrogen S.A.S., Sincelejo, Sucre, Colombia Pyrogen S.A.S. División de Investigación, Innovación y Desarrollo Pyrogen S.A.S. Sincelejo Sucre Colombia

**Keywords:** Lutzomyia evansi, Lutzomyia panamensis, Lutzomyia gomezi, multiplex polymerase chain reaction, cytochrome B, Lutzomyia evansi, Lutzomyia panamensis, Lutzomyia gomezi, reacción en cadena de la polimerasa múltiple, citocromo B

## Abstract

**Introduction.:**

El Alférez, a village in Los Montes de María (Bolívar, Colombia) and a macro-focus of leishmaniasis, recorded its first case in 2018, evidencing changes in the distribution and eco-epidemiology of the disease, although interactions between vectors and local fauna remain unknown.

**Objective.:**

To evaluate the diversity of sandflies and their blood meal sources in the community of El Alférez in the municipality of El Carmen de Bolívar (Bolívar, Colombia).

**Materials and methods.:**

In 2018**,** sandflies were collected using LED-based light traps in domestic, peridomestic, and sylvatic ecotopes and identified at the species level. Multiplex polymerase chain reaction targeting the mitochondrial cytochrome B gene was used to analyze blood from the digestive tract.

**Results.:**

*Lutzomyia evansi* was the most abundant species (71.85%; n = 485/675), followed by *Lu. panamensis*, *Lu. gomezi*, *Lu. trinidadensis*, *Lu. dubitans*, *Lu. abonnenci*, and *Lu.aclydifera.* Twenty-five percent of the species had blood meals from *Canis familiaris* (36.00%; n = 9/25), *Ovis aries* (36.00%; n=9:/25), *Bos taurus* (24.00%; n = 6/25)*, Sus scrofa* (20.00%; n = 5/25), and *Homo sapiens* (8.00%; n = 2/25). *Lutzomyia evansi* registered the highest feeding frequency (68.00%; n = 17/25), predominantly on a single (44.00%; n = 11/25) or multiple species (24.00%; n = 6/25).

**Conclusion.:**

Results indicate a eclectic feeding behavior in *Lu. evansi*, implying potential reservoir hosts for *Leishmania* spp. and increasing transmission risk. This study is a first step towards understanding the diversity of mammalian blood sources used by sandflies, that may be crucial for vector identification and formulation of effective control measures.

## Resumen

**Introducción.:**

En 2018, en la vereda El Alférez de Los Montes de María (Bolívar, Colombia), un macrofoco de leishmaniasis, se reportó el primer caso y se evidenciaron cambios en la distribución y ecoepidemiología de la enfermedad. No obstante, las interacciones entre vectores y fauna local aún son desconocidas.

**Objetivo.:**

Evaluar la diversidad de flebotomíneos y sus fuentes de alimentación sanguínea en la comunidad de El Alférez del municipio de El Carmen de Bolívar (Bolívar, Colombia).

**Materiales y métodos.:**

En el 2018, se recolectaron flebotomíneos mediante trampas de luz led ubicadas en el domicilio, el peridomicilio y en el área silvestre, y se identificaron a nivel de especie. Se utilizó la reacción en cadena de la polimerasa múltiple dirigida al gen mitocondrial citocromo B para analizar la sangre del aparato digestivo.

**Resultados.:**

*Lutzomyia evansi* fue la especie más abundante (71,85 %; n = 485/675), seguida por *Lu. panamensis*, *Lu. gomezi*, *Lu. trinidadensis, Lu. dubitans*, *Lu. abonnenci* y *Lu. aclydifera*. El 25 % (n = 25/100) de las especies analizadas tuvieron como fuentes de ingesta sanguínea a *Canis familiaris* (36 %; n = 9/25), *Ovis aries* (36 %; n = 9/25), *Bos taurus* (24 %; n = 6/25), *Sus scrofa* (20 %; n = 5/25) y *Homo sapiens* (8 %; n = 2/25). *Lutzomyia evansi* fue la especie con la mayor frecuencia de alimentación (68 %; n = 17/25), predominantemente de una sola especie (44 %; n = 11/25) o de varias (24 %; n = 6/25).

**Conclusiones.:**

Los hallazgos indican un comportamiento alimenticio ecléctico en *Lu. evansi* que implica potenciales reservorios para *Leishmania* spp. y eleva el riesgo de transmisión. Este estudio es un primer paso para comprender la diversidad de fuentes sanguíneas de mamíferos, utilizadas por los flebotomíneos, y que pueden ser cruciales para identificación de vectores y la formulación de medidas de control eficaces.

Phlebotomine sand flies (Diptera, Psychodidae: Phlebotominae) are vectors of *Leishmania* spp. (Euglenozoa, Kinetoplastea: Trypanosomatidae) and other trypanosomatids, bacteria, and arboviruses ^1^.

Historically, Los Montes de María in the Bolívar department has registered the highest visceral leishmaniasis incidence in Colombia, besides some cutaneous leishmaniasis cases. Between 2014 and 2019, 1,781 cases of cutaneous leishmaniasis and 54 of visceral leishmaniasis were reported in Bolívar ^2^. The wide temperature range (21-33 °C), 90% relative humidity, the abundant leaf litter, high rurality, and poverty, and a diverse peridomestic mastofauna would explain the high presence of phlebotomines and the 33.4% of visceral leishmaniasis cases in the tropical dry forest of El Carmen de Bolívar ^3^.

Numerous studies in Los Montes de María have focused on the identification of potential reservoirs of *Leishmania* spp. and other trypanosomatids ^4^^-^^11^, the diversity of phlebotomines, and the detection of natural infections with *Leishmania* spp. or other trypanosomatids ^6^^,^^9^^,^^12^^-^^28^. The sources of phlebotomine sand flies' blood intakes have also been examined ^27^^,^^29^^-^^31^.

Twenty phlebotomine sand fly species have been recorded in the department of Bolívar ^23^^,^^32^, among which the following stand out: *Lu. evansi* (Núñez-Tovar, 1924), incriminated as a vector of *Leishmania chagasi* (Cunha & Chagas, 1937) and *L. braziliensis* (Vianna, 1911), and visceral and cutaneous leishmaniasis etiologic agents, respectively; *Lu. gomezi* (Nitzulescu, 1931) vector of *L. braziliensis* and *L. panamensis* (David & Craft, 2009); and *Lu. panamensis* (Shannon, 1926), vector of *L. panamensis*, and the etiological agent of cutaneous leishmaniasis ^27^^,^^33^.

Other studies on Bolívar’s phlebotomines have identified 19,649 sand flies, with *Lu. evansi* having the highest abundance ^4^^,^^7^^,^^14^^,^^20^^,^^21^^,^^28^.

Another study reported *Lu. evansi* females feeding on *H. sapiens* (16.73%; n = 82/490), *Capra hircus (*16.32%; n = 80/490), *S. scrofa* (12.45%; n = 61/490), *B. indicus* (11.63%; n = 57/490) and *C. familiaris* (9.79%; n = 48/490) ^30^. In other areas in Los Montes de María, polymerase chain reaction (PCR) has also been used as a sensitive technique to identify groups of mammals serving as blood sources for *Lutzomyia*^29^^,^^30^. The study of phlebotomine involved in the transmission of *Leishmania* spp. and its blood sources in El Carmen de Bolívar may increase our knowledge regarding domestic and peridomestic ecotopes in an area of high visceral leishmaniasis endemicity in Colombia and some reported cutaneous leishmaniasis cases.

In this context, the present study evaluated sand fly diversity in El Alférez village, located in the municipality of El Carmen de Bolívar (Bolívar) in northern Colombia, in the hope of establishing a molecular approach for blood sources identification through analysis of sand flies digestive tract.

## Materials and methods

The study was conducted in El Alférez, a rural area endemic for both visceral and cutaneous leishmaniasis in El Carmen de Bolívar municipality (09° 45' 38" N; 075° 10’ 19.1" W), department of Bolívar, in northern Colombia. It is a tropical dry forest with bimodal rainfall (May-June with 68 mm^3^ and September-November with 104 mm^3^). The landscape includes patches of fruit trees such as cocoa and avocado, and timber trees ^23^. We collected the phlebotomines in four samplings from January to November 2018, using led-based light traps placed in the domestic, peridomestic, and sylvatic surroundings after the household heads signed the informed consent. We defined a sylvatic ecotope as an area over 100 m in diameter from the dwelling. Trapping was done on three consecutive nights from 18:00 to 06:00 hours for a trapping effort of 1,728 hours.

We carefully preserved females with evidence of blood remnants in the abdomen in Eppendorf® microtubes. We isolated the last three abdominal segments, cleared them in lactophenol (1:1), and carefully examined in the microscope (400X). Taxonomic keys and reference images were used to analyze morphometric and anatomical characters ^34^, and 100 fattened females were randomly selected for DNA extraction using the salting out method described in a previous work ^35^. We performed a multiplex conventional PCR assay to identify mixed blood sources in phlebotomines associated with human dwellings, minimizing problems related to the universality of primers that could amplify the sand flies genome or multiple vertebrate species. Then, we quantified the extracted DNA and amplified mitochondrial cytochrome B (*Cytb*) fragment. The targeted species-specific amplicons included *O. aries* (132 bp), *H. sapiens* (334 bp), *S. scrofa* (453 bp), *B. taurus* (561 bp), and *C. familiaris* (680 bp); we used the primers described in other studies ^36^.

The final PCR mixture had a final volume of 12 μl with 4 μl of target DNA, 10 μl of reaction mixture containing GoTaq® Green Master Mix 2X (Promega, Madison, USA), 10 μΜ of primer mix, and ultrapure water. We set 58 °C as the melting temperature, estimated based on each primer pair's average temperatures minus five Celsius degrees. The rest of the profile was programmed following the manufacturer’s specifications. We analyzed the PCR products running a 2% agarose gel in TBE (tris-borate-EDTA) buffer supplemented with SYBR Green. Finally, we estimated amplicon sizes by comparing them with a 100-1,500 bp DNA ladder (Thermo Fisher Scientific, CA).

All specimens were collected under the global permit in Resolution 0391, April 11, 2016, granted by the national environmental licensing authority to the *Universidad de Sucre*. We conducted animal experiments following the guidelines outlined in Resolution 008430 of 1993 and 2.378 of 2008, issued by the Colombian *Ministerio de Salud y Protección Social,* establishing the scientific, technical, and administrative research norms. The project was approved by the bioethics committee of *Universidad de Sucre* (Meeting 07, 2019) in compliance with the relevant resolutions expedited by the higher and academic councils at *Universidad de Sucre.*

## Results

We collected 675 specimens belonging to the *Lutzomyia* genus and identified seven species: *Lu. evansi* (71.85%; n = 485/675), *Lu. gomezi* (15.55%; n = 105/675), *Lu. panamensis* (4.74%; n = 32/675), *Lu. aclydifera* (Fairchild & Hertig, 1952) and *Lu. trinidadensis* (Newstead, 1922) (0.30% each; n = 2/675), *Lu. dubitans* (Sherlock, 1962) and *Lu. abonnenci* (Floch & Chassignet, 1947) (0.14% each; n = 1/675). Forty-seven (6.96%) individuals were not identified.

The highest abundance and richness of species were registered in peridomestic (44.00%; n = 297/675) and sylvatic (85.71%; n = 6/7) ecotopes, while the lowest ones were in ecotope D (table 1).

**Table 1:** Diversity and abundance of phlebotomines collected in El Alférez, El Carmen de Bolívar (Bolívar), in northern Colombia

Species	D	PD	S	Total (%)	
* *Lutzomya evansi*	112	216	157	485	(71.85)
* *Lutzomya gomezi*	31	50	24	105	(15.55)
* *Lutzomya panamensis*	1	14	17	32	(4.74)
*Lutzomya trinidadensis*	0	1	1	2	(0.30)
*Lutzomya dubitans*	0	1	0	1	(0.15)
*Lutzomya aclydifera*	0	0	2	2	(0.30)
*Lutzomya abonnenci*	0	0	1	1	(0.15)
*Lutzomyia* spp.	17	15	15	47	(6.96)
Total	161	297	217	675	(100)

D: Domestic; PD: Peridomestic; S: Sylvatic

* Species of epidemiological importance in Leishmania transmission

**Figure 1.**
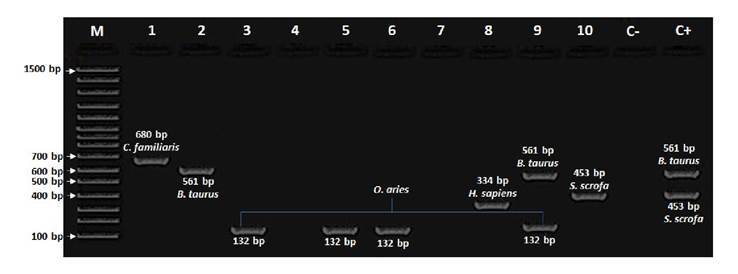
Agarose gel electrophoresis (2%; 80 V for 70 minutes) showing amplicons of mitochondrial cytochrome B gene, isolated from sand flies intestinal content collected in El Alférez, a rural area of El Carmen de Bolívar (Bolívar), in northern Colombia. Lane 1: *Lutzomyia evansi* DNA (amplicon for *Canis familiaris*); Lane 2: *Lutzomyia evansi* DNA (amplicon for *Bos taurus*); Lanes 3, 5, 6: *Lutzomyia gomezi* DNA (amplicons for *Ovis aries*); Lanes 4, 7: *Lu. evansi* DNA (no amplicon present); Lane 8: *Lu. evansi* DNA (amplicon for *Homo sapiens*); Lane 9: *Lu. gomezi* DNA (amplicons for *Bos taurus* and *Ovis aries*); Lane 10: *Lu. evansi* DNA (amplicon for *Sus scrofa*); C-: Non-template control; C+: Positive control (mixture of genetic material from *Bos taurus* and *Sus scrofa*).

*Cytb* PCR from the genetic material of the sand flies digestive tract showed amplicons for a single and multiple blood meals in 25.0% of the specimens (25/100) (figure 1).

M: Molecular marker (100-1,500 pb).

*Canis familiaris*, *H. sapiens*, *B. taurus*, *S. scrofa*, and *O. aries* appeared as blood sources; *C. familiaris* blood was present in 66.0% (6/9) of the mixed blood meals and in 18.7% (3/16) of the single blood meals.

*Lutzomyia evansi* (17 individuals fed) showed the most generalist feeding habits with single and mixed blood meals, followed by *Lu. gomezi* (six individuals fed) with five single intakes and one mixed blood meal, and *Lu. panamensis* with only two mixed intakes (table 2).

**Table 2:** Mixed and single blood meals for medically important phlebotomine species collected in El Alférez, El Carmen de Bolívar (Bolívar), in northern Colombia

Species	Mixed bloodmeals	n	Single bloodmeals	n
*Lutzomyia evansi*	*Ovis aries/Sus scrofa/Canis familiaris*	1	*Canis familiaris*	3
	*Ovis aries/Canis familiaris*	2	*Homo sapiens*	1
	*Canis familiaris/Bos taurus*	2	*Ovis aries*	5
	*Ovis aries/Sus scrofa/Bos taurus*	1	*Bos taurus*	1
			*Sus scrofa*	1
*Lutzomyia gomezi*	*Sus scrofa /Bos taurus*	1	*Ovis aries*	5
*Lutzomyia panamensis*	*Sus scrofa/Homo sapiens*	1	-	-
	*Bos taurus/Canis familiaris*	1	-	-
Total		9		16

## Discussion

The abundance and diversity of the sand flies collected in El Alférez, Los Montes de María, responds to this region being a tropical dry forest corridor with deciduous trees, copious organic matter from decomposing litter, alkaline soils, and little human intervention, a suitable habitat for phlebotomines ^26^^,^^37^. Besides, the plentiful domestic and synanthropic fauna are fitted for the *Lutzomyia* spp. life cycle ^38^.

*Lutzomyia* has a wide distribution in the country, with 20 species recorded in the department of Bolívar _^(23,32)^_ , seven (35.0%) of which were found during our study. The composition and abundance patterns of the phlebotomines found are similar to those found in previous research in the Colombian Caribbean, with *Lu. evansi* dominating, followed by *Lu. gomezi,* and *Lu. panamensis*^6^^,^^9^^,^^12^^-^^16^^,^^18^^,^^19^^,^^21^^-^^23^^,^^26^^,^^27^^,^^39^^,^^40^. *Lutzomyia evansi* high abundance has been registered in previous studies in Colombia's northern region, including the departments of Atlántico (74.92%) (^39^,^40^), Córdoba (86.38%) ^12^^-^^14^^,^^26^^,^^27^, Sucre (90.80%) ^15^^,^^18^^,^^19^^,^^21^^,^^26^, and Bolívar (64.66%) ^6^^,^^16^^,^^22^^,^^23^.

The significant presence of *Lu. evansi* suggests a remarkable species’ adaptability to highly anthropized environments because of its varied diet ^26^. Furthermore, the loss and fragmentation of the original habitat due to agricultural activities, a characteristic of the tropical dry forest, may be associated with this species ^39^.

Our results show that *Lu. evansi* uses both *B. taurus* and *C. familiaris* as blood sources, as recorded elsewhere in the country, particularly in the department of Sucre ^29^. This behavior correlates closely with the intense livestock activity in the Colombian Caribbean because *Lu. evansi* would have more opportunities to use *B. taurus* as a highly available blood source ^29^. Although cattle’s role in the epidemiological cycle of leishmaniasis in Colombia has been traditionally underestimated, studies in Brazil have shown that *L. infantum* can infect *B. taurus*^41^, suggesting a possible link between livestock practices and leishmaniasis transmission and highlighting the need to investigate the role of these domestic animals in disease dynamics.

We compared our evidence regarding *Lu. evansi* feeding on pigs (*S. scrofa*) with that from other authors ^29^ and concluded that *Lu. evansi* zoo-anthropophilic habits and high abundance in domestic and peridomestic ecotopes, where pig farms are usually located, constitute a risk scenario for *Leishmania* spp*.* transmission.

Blood ingestion from *O. aries* has been found in *Lu. evansi*. However, sheeps have not a clear role as a host for *Leishmania* spp. but are very common in the peridomestic. Also, phlebotomines lay their eggs in soils rich in organic matter, mainly due to pig, sheep, and cattle farming, which provides nutrients for *Lutzomyia* spp. larval instars ^38^.

A single *Lu. evansi* specimen showed *H. sapiens* blood DNA traces in its intestinal content, in contrast with the high frequency of this feeding source in individuals from urban areas in the department of Sucre and Los Montes de María rural areas ^12^^,^^27^^,^^28^^,^^30^.

*Lutzomyia evansi* multiple intakes and probable blood source variety would ensure *Leishmania* spp. transmission to mammals -including humans- in domestic, peridomestic, and sylvatic surroundings. However, the few individuals fed with *H. sapiens* may indicate a limitation for zoonotic scenarios, but this requires further studies.

*Lutzomyia gomezi,* the second most abundant species recorded in this study, is an epidemiologically relevant species in Colombia distributed in 28 of its 32 departments ^32^ and reported to be infected with the cutaneous leishmaniasis agents: *L. panamensis* and *L. braziliensis*^27^.

We emphasize that although the role of *O. aries* in the transmission cycle of *Leishmania* is unknown, it cannot be excluded as a potential reservoir since five individuals presented *O. aries* DNA traces*.*

Despite low frequency in peridomestic and sylvatic areas, *Lu. panamensis* is known for its importance in medical entomology. It is present in 14 Latin American countries and has been found in 22 out of 32 departments of Colombia ^32^. *Lutzomyia panamensis*, considered highly anthropophilic, is the primary vector of *L. panamensis,* a cutaneous leishmaniasis etiological agent in Colombia ^27^.

The finding of *H. sapiens* as a blood source of *Lu. panamensis*, already referred to in northern Colombia (Sucre and Córdoba), would probably evidence an eventual anthropophilic habit ^27^^,^^29^.

Here, we had no results for intake sources in 75.00% (n = 75/100) of the individuals, which may be explained by prolonged fasting as females were fertilized but not ready to oviposit or were feeding on other wildlife vertebrates not evaluated in our study.

*Lutzomyia trinidadensis*, a species with a wide distribution due to its presence in 15 Latin American countries and 20 out of 32 Colombian departments ^32^, was among the less frequent species found in this sampling, perhaps because we collected the sand flies during the rainy season when this species has a low abundance. In Venezuela, Bonfante-Garrido *et al.*^42^ found promastigotes in *Lu. trinidadensis* and inoculated them into hamsters, showing tissue invasion by amastigotes morphologically compatible with *L. venezuelensis.* This finding suggested that *Lu. trinidadensis* is a potential vector of *Leishmania* in that country, highlighting the need to monitor its populations in Colombia.

*Lutzomyia abonnenci* is relevant given its widespread distribution in Bolivia, Brazil, Ecuador, French Guiana, Panamá, Perú, Suriname, Venezuela, and Colombia, where it has a low frequency in 11 out of 32 departments, including Bolívar ^32^, and, so far, no role in *Leishmania* spp. transmission.

*Lutzomyia dubitans*, the other species found in the study, has been reported in Brazil, Costa Rica, Panama, Trinidad and Tobago, and 16 out of 32 departments of Colombia ^32^, but its role in *Leishmania* spp. transmission is uncertain.

*Lutzomyia aclydifera* of the *dreisbachi* group is a sparsely distributed species in Colombia, with records from Antioquia, Chocó, Valle del Cauca, and, more recently, Bolívar ^23^^,^^32^. As with *Lu. abonnenci* and *Lu. dubitans*, its role as a vector for *Leishmania* spp. is unknown*.*

Although we did not intend to identify all mammalian species as blood sources, the technique allowed the detection of the most abundant nourishment sources among domestic species in areas close to human dwellings ^27^^,^^29^^-^^31^. In this sense, sequencing techniques would provide higher accuracy in identifying blood sources. However, they would also prevent the identification of mixed blood sources and the sand flies’ sequences ^30^.

Finally, multiplex PCR for domestic vertebrates DNA detection allowed us to identify the blood meals of three species with high epidemiological value, *Lu. evansi, Lu. gomezi,* and *Lu. panamensis*, which fed on *B. taurus*, *H. sapiens*, *C. familiaris*, *O. aries,* and *S. scrofa* (domestic and peridomestic fauna).

This report is the first approach to mammals serving as blood sources and acting as possible hosts/reservoirs of *Leishmania* spp. in El Carmen de Bolívar. The presence of this phlebotomine-mammalian binomial in domestic and peridomestic areas could represent a transmission risk.
